# Dose-escalation of oxaliplatin in hemodialysis patient treated with FOLFOX therapy: A case report

**DOI:** 10.1097/MD.0000000000017462

**Published:** 2019-11-01

**Authors:** Danping Wang, Xiaofei Li, Lingyan Xu, Wentong Fang, Xiaomin Cai, Ying Wang, Jiawei Wang, Yuanyuan Wang, Fengjiao Zhao, Yanhong Gu

**Affiliations:** a Department of Oncology; b Department of Pharmacy, The First Affiliated Hospital of Nanjing Medical University, Nanjing, China.

**Keywords:** FOLFOX, hemodialysis, oxaliplatin, pharmacokinetics

## Abstract

**Rationale:**

Oxaliplatin is a key part of the standard treatment for colorectal cancer which is formally contraindicated in patients with severe renal dysfunction. Here, we investigated a safe and efficient dosing schedule of oxaliplatin in folinic acid, fluorouracil, and oxaliplatin (FOLFOX) regimen by monitoring total and free platinum concentrations in plasma.

**Patient concerns:**

A 47-year-old female with chronic hemodialysis was diagnosed with left-sided colon cancer and underwent colectomy. One year later, she was presented with omentum metastasis and needed further treatment.

**Diagnoses:**

The computed tomography (CT) scanning revealed multiple omental nodules. Positron emission tomography-CT (PET-CT) showed increased uptake of the nodules.

**Interventions:**

The patient was treated with FOLFOX therapy every 3 weeks. The oxaliplatin began with 50 mg/m^2^ and gradually increased 85 mg/m^2^ as in the standard regimen. A 4-hour dialysis was started 1 hour after the end of oxaliplatin infusion.

**Outcomes:**

The free platinum concentration time curve showed a biomodel pattern. The *C*_max_ of the 1st peak we observed in our patients at the standard dose is comparable to patients with normal renal function. This patient was treated with FOLFOX for 12 courses. No apparent adverse effect was observed during the treatment.

**Lessons:**

The FOLFOX can be safely administered in hemodialysis patients on a long-term basis. Dose reduction of oxaliplatin is not necessarily needed if hemodialysis is performed soon after the infusion. Further studies are needed to distinguish between active and inactive oxaliplatin products during the 2nd peak of the free platinum concentration curve in this population.

## 1 Introduction

With the improvement of renal replacement therapy, the number of patients undergoing chronic hemodialysis has been increasing in recent years. However, dialysis patients are under a higher risk of cancer compared with the general population.^[1]^ FOLFOX is a chemotherapeutic regimen that consists of oxaliplatin, 5-fluorouracil, and levofolinate and is widely used as one of the standard treatment for metastatic colorectal cancer. Oxaliplatin is mainly eliminated through the kidney. Its elimination is delayed in patients with renal insufficiency and limited data are available on the pharmacokinetics, efficacy and safety of oxaliplatin in hemodialysis patients.^[2]^ To date, only a few case reports of dosing oxaliplatin in hemodialysis patients have been published.^[3–7]^ In this study, we report the case of a hemodialysis patient with metastatic colon cancer who was safely treated with FOLFOX therapy by monitoring total and free platinum concentrations in plasma.

## 2 Case report

A 47-year-old female with chronic glomerulonephritis had been maintained on hemodialysis since 2012. In April 2016, she was diagnosed with left-sided colon cancer and underwent colectomy. The pathologic stage is IIA (T3N0M0) without any high-risk factors, so she has not received adjuvant chemotherapy. In March 2017, the follow-up computed tomography (CT) scanning revealed multiple omental nodules. Positron emission tomography-CT (PET-CT) showed increased uptake of the nodules.

The patient was treated with reference to the modified FOLFOX protocol, given every 3 weeks. Oxaliplatin and levofolinate (200 mg/m^2^) were infused simultaneously for 2 hours. 5-Fluorouracil was then administered as a bolus injection of 400 mg/m^2^, followed by continuous infusion of 2400 mg/m^2^ for 46 hours. The dose of oxaliplatin was started with 50 mg/m^2^ (58% of the standard dose of 85 mg/m^2^) in the 1st cycle and increased to 60 mg/m^2^ in the 2nd cycle. And the dose was increased to 70 mg/m^2^ in the next 3 cycles. Since no obvious adverse effect was observed, the standard 85 mg/m^2^ was used for the following cycles. Most of 5-fluorouracil is eliminated by hepatic metabolism and only 10% was excreted by kidney. Previous studies have shown that dose adjustment of 5-fluorouracil is not needed for patients undergoing hemodialysis.^[5,8,9]^ A 4-hour hemodialysis session was started 1 hour after the administration of oxaliplatin with a polysulfonate hollow fiber dialyzer (REXEED-15L). The blood flow rate was 200 mL/min and the dialysate flow rate was 500 mL/min.

**Figure 1:**
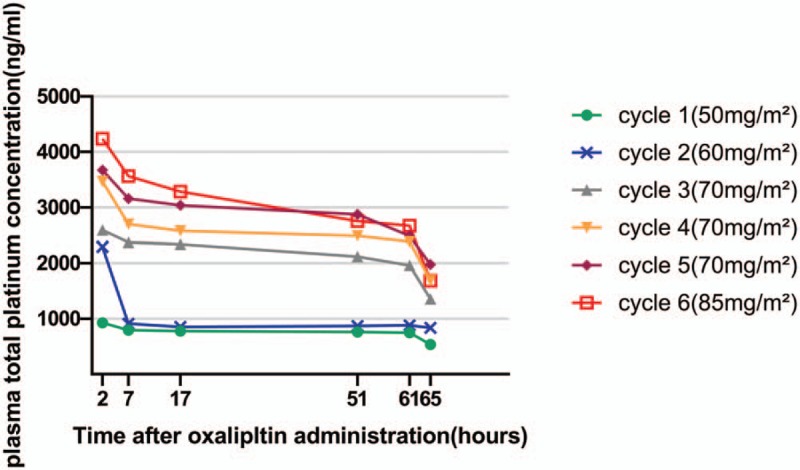
Total platinum in plasma concentration time curve.

**Figure 2:**
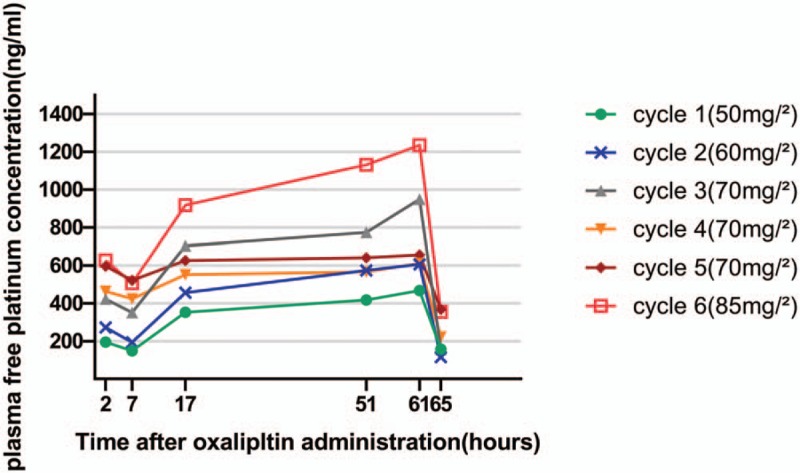
Free platinum in plasma concentration time curve.

Total and free platinum levels were monitored during the first 6 cycles. We collected the blood samples at the following time points: 2 (immediately after the administration of oxaliplatin), 7 (after the 1st hemodialysis), 17, 51, 61 (before the 2nd hemodialysis), and 65 (after the 2nd hemodialysis) hours after the beginning of oxaliplatin infusion. Each blood sample was centrifuged at 3500 rpm for 5 minutes and the plasma thus obtained was further centrifuged at 15,000 rpm for 10 minutes in an ultrafiltration tube. Then the plasma and ultrafiltrate samples were assayed of the platinum content by inductively coupled plasma mass spectrometry (ICP-MS, 7700X; Agilent Technologies, Santa Clara, CA). Table 1 shows the *C*_max_ data for free platinum in plasma. Figures 1 and 2 show the time course of total and free platinum levels, respectively.

**Table 1:**
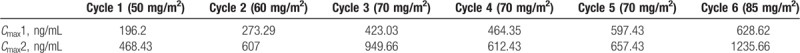
Pharmacokinetic parameters of plasma free platinum.

In all, 12 courses of FOLFOX therapy were completed. The follow-up CT scans at 6 and 10 months showed stable disease. The patient did not develop bone marrow suppression during the treatment. The total dose of oxaliplatin reached 1455 mg and no symptoms of peripheral neuropathy was observed. In July 2018, CT scan showed increased size of the tumors and she underwent abdominal tumor excision and postoperative chemotherapy.

## 3 Discussion

At the end of infusion, the majority (approximately 73%) of the administered platinum rapidly binds to plasma proteins and erythrocytes or be eliminated in the urine.^[2]^ The remaining platinum in the systemic circulation is thought to represent the antitumor activities and side effects of oxaliplatin.^[10,11]^ Renal excretion is the major route of platinum elimination. The renal clearance of oxaliplatin is correlated with glomerular filtration rate and glomerular filtration is a primary mechanism of platinum clearance. According to the findings of Watayo et al,^[4]^ free platinum can be completely eliminated by hemodialysis.

In this case, the free platinum concentration time curve showed a biomodel pattern, which is consistent with the previous studies.^[4–7]^ The 2nd peak was even higher than the 1st one and was showed just before the 2nd dialysis session. The free platinum level decreased during the 1st hemodialysis and rose gradually until the next dialysis session. Such a 2nd peak was not seen in patients with normal renal function which indicates the drug exposure was strongly increased in the hemodialysis patients. During the 2nd dialysis session, the free platinum level decreased rapidly again and reached a relatively low level in this patient. According to a previous report, the free platinum level would remain low until the 3rd dialysis and can be eliminated by repeated hemodialysis.^[4]^

The kinetics of free platinum can be well described as triexpontential, characterized by a short alpha-phase, a longer beta-phase, and a very long gamma-phase.^[2]^ The alpha-phase has a half-life of 0.28 hours. It represents the rapid clearance of the active platinum by binding to plasma proteins and erythrocytes. The beta-phase has a half-life of about 16.3 hours and represents the renal clearance of free platinum, which can only be eliminated by hemodialysis in our patient. Former studies indicated that for patients with impaired renal function, the area under the curve of free platinum was increased but toxicity was not accordingly increased.^[12–14]^ That might be due to oxaliplatin is quickly converted into inactive conjugates after the infusion. Thus, Takimoto et al hypothesized that the platinum eliminated in this phase is mostly consist of low-molecular-weight conjugates which are inactive and not correlated with toxicity. The long gamma-phase has a half-life of 273 hours. This phase represents the slow release of platinum from degradation of cellular macromolecules. The increasing level of platinum after the 1st dialysis is likely due to the dissociation of platinum bound to plasma proteins and erythrocytes in the alpha-phase.^[15]^ However, it is still not clear whether this part of platinum has biological activity. Although the exposure of oxaliplatin is significantly increased due to the 2nd peak, no increased toxicity was observed in our patient. It is likely that the 2nd peak comprised almost entirely of inactive platinum conjugates which do not increase the toxicity of oxaliplatin.

Previous study showed that patients with insufficient renal function have comparable *C*_max_ values to those with normal renal function.^[12]^ The *C*_max_ of the 1st peak we observed in our patient at the standard dose (628 ng/mL) is also comparable to patients with normal renal function (681 ng/mL).^[2]^ It is possible that the active forms of oxaliplatin are eliminated shortly after the infusion by clearance mechanisms independent of renal function. Thus, the *C*_max_ of the 1st peak might be more clinically relevant.

Considering whether the increased platinum exposure from the 2nd peak will increase the risk of adverse events is still unclear, the 3-week dosing interval may be optimal for this patient.

In this study, we reported a woman on chronic hemodialysis who was safely treated with FOLFOX therapy for nearly a year. Based on our data, dose reduction in oxaliplatin is not necessarily needed if hemodialysis is performed soon after the administration of oxaliplatin and the dosing interval is increased to 3 weeks. Further studies are required to distinguish between active and inactive oxaliplatin products during the 2nd peak of the free platinum concentration curve in hemodialysis patients to optimize the oxaliplatin dose and timing of dialysis in this population.

## Author contributions

**Conceptualization:** Yanhong Gu.

**Data curation:** Xiaofei Li, Xiaomin Cai.

**Methodology:** Wentong Fang, Fengjiao Zhao.

**Resources:** Ying Wang, Jiawei Wang, Yuanyuan Wang.

**Writing – original draft:** Danping Wang, Xiaofei Li.

**Writing – review & editing:** Lingyan Xu.
